# Rapid and Sensitive Determination of Methylxanthines in Commercial Brands of Tea Using Ultra-High-Performance Liquid Chromatography-Mass Spectrometry

**DOI:** 10.1155/2019/2926580

**Published:** 2019-11-03

**Authors:** Ahmad Aqel, Ahmed Almulla, Asma'a Al-Rifai, Saikh M. Wabaidur, Zeid A. ALOthman, Ahmed-Yacine Badjah-Hadj-Ahmed

**Affiliations:** ^1^Department of Chemistry, College of Science, King Saud University, P.O. Box 2455, Riyadh 11451, Saudi Arabia; ^2^Department of Chemistry, College of Science, Princess Nourah Bint Abdulrahman University, Riyadh, Saudi Arabia

## Abstract

Recently, chromatographic techniques have the potential to be greener in order to reduce the environmental impact. In this work, a new simple, sensitive, efficient, and green analytical method based on UHPLC-MS has been developed for a quick determination of methylxanthines including caffeine, theobromine, and theophylline in tea. Under the optimum conditions, a baseline separation has been achieved within 30 seconds, using isocratic elution consisting of 90% water and only 10% acetonitrile at 0.5 mL/min flow rate (3 mL acetonitrile per hour). The mass spectrometer was operated with the SIR mode in ESI^+^. The developed method was found to be linear in the range of 0.03–5 *μ*g/mL, with correlation coefficients greater than 0.9995 for the three compounds. The respective values of LOD were found to be 0.025, 0.015, and 0.01 *μ*g/mL for caffeine, theobromine, and theophylline, respectively. The proposed assay was applied to 30 commercial tea samples of different brands. Both caffeine and theobromine were found in all tea samples with maximum concentration in sample no. 15, corresponding to 32.6 and 2.72 mg/g of caffeine and theobromine, respectively. On the contrary, theophylline was not detected at all in most samples. When compared with all previous studies that dealt with the same compounds in different matrices, the developed method was found to be the fastest, allowing high-throughput analyses with more than 100 samples/h. The results prove that the method is suitable for routine analysis of methylxanthines and to distinguish the quality of tea samples of various brands.

## 1. Introduction

Nowadays, special interest has been paid to tea extracts because they are the most popular and oldest beverages used all around the world with the exception of water [1]. Tea is classified into fully fermented as in black tea, semifermented as in oolong tea, slightly fermented as in white tea, and unfermented as in green tea [2, 3]. These tea types are originally prepared from the leaves of *Camellia sinensis* L. (Theaceae family) [4]. Several studies have demonstrated the beneficial health effects of tea leaves extract due to specific substances found in it [5], such as caffeine, catechins, amino acids, fats, vitamins, chlorophyll, and phenolic compounds [6, 7]. Tea leaves extract may play an important role to prevent chronic gastritis, cardiovascular disease, and arteriosclerosis [8]. In addition, it has antioxidant and anticancer activities [9].

Methylxanthines have various physiological effects on the body, including stimulatory effects on the central nervous and respiratory systems [10, 11], bronchial muscle relaxing [11], and gastrointestinal, cardiovascular [12], and myocardial stimulation [13], with increasing blood pressure [14]. They have been used as a therapeutic agent for treatment of asthma [8] and as antitussive [11], diuretic [15], and antimigraine [16]. On the other hand, high amounts of these substances in human body may cause depression and hyperactivity [17], increased alertness, insomnia, and improvements in learning capacity and exercise performance. They can also induce reduction of fatigue [18], headaches, dizziness, muscle pain, nervousness, nausea, trembling, and seizure [19], with negative effects on premenstrual syndrome and pregnancy, cancer [4], anorexia, and heartburn tachycardia [16]. A fatal dose of caffeine has been reported to be about 170 mg per kg of body weight, and the caffeine overdose is more than 200 mg per day [16, 19, 20].

Caffeine (1,3,7-trimethylxanthine), theobromine (3,7-dimethylxanthine), and theophylline (1,3-dimethylxanthine) are the most important naturally occurring methylated xanthine alkaloids found in tea leaves [1, 21]. The structural differences between these compounds are clear in the number and position of methyl substituents in the xanthine ring. While theobromine and theophylline are dimethylxanthine isomers, and caffeine corresponds to the trimethyl-substituted homologue. The similarity in the chemical structures of these compounds makes their simultaneous separation and analysis difficult.

A variety of analytical techniques and sample preparation procedures have been developed and registered for determination and separation of these three methylxanthines in plants, environmental samples, drugs, food, and biological fluids. These proposed methods include UV-Vis spectrophotometry [22–24], thin-layer chromatography (TLC) [25], ion chromatography (IC) [26], FT-Raman spectrometry [27], IR spectrometry [28], capillary electrophoresis (CE) [29, 30], NMR spectroscopy [31], mass spectroscopy [32], gas chromatography-mass spectrometry (GC-MS) [15], high-performance thin-layer chromatography (HPTLC) [33, 34], voltammetry [35, 36], and high-performance liquid chromatography (HPLC) which was reported as the most frequently used approach [1, 2, 10, 37–49].

Considering the discussions above, the present work was intended to develop and validate a new methodology for the determination of caffeine, theobromine, and theophylline in tea samples using reversed phase ultra-high performance liquid chromatography with MS detection. The developed method was compared with all previous reports used for analysis of one or more of methylxanthines in plants, drugs, food, and other samples. To the best of our knowledge, determination of caffeine, theobromine, and theophylline by UHPLC-MS has not been previously reported in the literature.

## 2. Materials and Methods

### 2.1. Standards, Reagents, and Tea Samples

Caffeine, theobromine, and theophylline were purchased from Sigma (St. Louis, MO, USA). A total of 30 tea samples of different brands (provided in Table 1) were purchased from local supermarkets (Riyadh, Saudi Arabia) as powder and stored in vacuum packages at a temperature below 0°C. HPLC grade acetonitrile and formic acid were purchased from BDH (Lutterworth, UK). Water used as the mobile phase solvent was prepared using a Millipore system (Milli-Q Advantage Elix, Millipore S.A.S. 67120 Molsheim, France), and then filtered on 0.2 *μ*m nylon membrane filter from Whatman (Maidstone, UK).

Stock solutions of caffeine, theobromine, and theophylline were 100 *μ*g/mL, and they were prepared by dissolving the standard of each compound with an appropriate amount of water and subsequently stored at 4°C. The working standards were prepared by diluting the concentrated stock standard solutions in water before experiment. A calibration curve was constructed each day before analysis of the samples. HPLC grade water was injected as the blank.

### 2.2. Sample Preparation and Extraction Procedure

Thirty commercial samples of tea were ground to a fine powder. In order to simulate the preparation of tea in the most common method, an accurately weighed amount of 100 mg of each individual tea sample was extracted with 50 mL water in a round-bottom flask for 5 min with the temperature maintained at 80°C. The resulting mixtures were then filtered through 0.22 *μ*m nylon filters, and each filtrate was diluted and injected into the UHPLC system under the optimum conditions without further pretreatment. The tea samples were reextracted under the same conditions to test for quantitative extraction; however, no measurable peaks were recorded at the retention time of the three analytes for the most studied samples.

### 2.3. UHPLC Conditions

The chromatographic analyses of the standards caffeine, theobromine, and theophylline as well as all real samples were performed on an Acquity UPLC system (Waters Corp., Milford, MA, USA), including an Acquity UPLC binary solvent manager, a sample manager, and a column heater.

The liquid chromatographic separations of all analyzed samples were achieved under isocratic conditions using Nucleodur C_18_ polar column Tec. (50 × 2 mm i.d.; particle size: 1.8 *μ*m) from Macherey-Nagel (Düren, Germany). The column temperature was kept at 70°C, and the sample manager was maintained at 10°C. A binary mixture composed of acetonitrile/water (10 : 90, v/v) with 0.1% formic acid at a flow rate of 0.5 mL/min was employed as the mobile phase. Peak area was used for quantitative evaluations. All standard and real samples were injected five times. Standard deviations and statistical parameters were calculated using Microsoft Office Excel software 2013 package. The sample injection volume was fixed at 5.0 *μ*L.  Table 2 summarizes the optimum conditions of UHPLC. Due to the presence of many different ingredients in the tea such as catechins, polyphenols, flavonoids, and amino acids, the late-eluted compounds from the sample matrix (which are undetectable under the stated MS conditions) were washed with acetonitrile and water after analysis of each tea sample.

### 2.4. MS Measurements

A Quattro Premier triple-quadrupole mass spectrometer equipped with the electrospray ionization (ESI) source of Micromass Company Inc. (Manchester, UK) was used for mass spectrometry measurements. The ESI source was used for ionization of target compounds. A SOGEVAC SV40 BI Oerlikon rotary pump (Paris, France) produced the significant vacuum in the mass spectrometer. All the mass spectrometry measurements have been performed using positive electrospray ionization (ESI^+^) with the selective ion reaction monitoring (SIR) mode. All experimental conditions were optimized for achieving the highest peak intensity. The specific cone voltage was optimized for the formation of parent ions. High-purity nitrogen gas created by a nitrogen generator of Peak Scientific NM30LA (Inchinnan, UK) was supplied to the ion source for nebulizing purpose. All experimental data collection and processing were carried out by using MassLynx V4.1 software (Micromass, Manchester, Lancashire, UK). All MS parameters optimized in this work are listed in Table 2.

### 2.5. Validation

The developed method was validated in terms of linearity, limits of detection (LOD) and quantitation (LOQ), precision, ruggedness, and recovery tests. External calibration curves have been constructed for each analyte directly before analysis of the real tea samples. Concentrations ranging from 0.05 to 3 *μ*g/mL of the three compounds were obtained for the standard solutions. The solutions were always filtered using a 0.22 *μ*m filter before injection, and each solution was injected in five replicates. In order to study the method linearity, peak areas were plotted versus the respective concentrations of caffeine, theobromine, and theophylline, individually. LOD and LOQ values of the three compounds corresponded to concentrations that lead to signal-to-noise ratios of 3 : 1 and 10 : 1, respectively.

In order to study the efficiency of the extraction at the optimal point, recovery tests were performed. As a model, sample no. 5 was spiked with standard solution of the three solutes at three concentration levels: 0.1, 1.0, and 3.0 *μ*g/mL.

## 3. Results and Discussion

### 3.1. Optimization of UHPLC Conditions

To obtain the best peak resolution and to minimize the peak tailing of the target compounds, the liquid chromatographic parameters including column type and size, column temperature, composition, and flow rate of the mobile phase were optimized. Full separation of caffeine, theobromine, and theophylline was achieved by using UHPLC-MS on a Nucleodur C_18_ polar Tec. Macherey-Nagel column (50 × 2 mm id; 1.8 *μ*m particle sizes) with an isocratic elution mode of a binary mobile phase mixture of acetonitrile and water (10 : 90) with 1% formic acid (v/v) at a constant flow rate of 0.5 mL/min. In order to lower the back pressure of the column, its oven temperature was tested for better separation of the analytes in the range 25–90°C, and the best results were obtained at 70°C and for an injection volume of 5.0 *μ*L. All UHPLC conditions are listed in Table 2. These conditions made possible to achieve an analysis time of about 30 s with a sampling rate exceeding 100 samples/h. The retention times obtained were 14, 19, and 28 s for theobromine, theophylline, and caffeine, respectively; these values correspond to the shortest analysis time reported in the literature for the studied compounds [1, 2, 10, 13, 15, 19, 20, 37–49], and some examples are given in Table 3.

In comparison with the other techniques used for analysis of these methylxanthines in different samples [15, 22–36], the developed method showed high sensitivity and very short analysis time allowing high-sample throughput. On the other side, techniques such as UV-vis spectrophotometry, FT-Raman spectrometry, IR spectrometry, and NMR spectroscopy are not sensitive and unfavorable for analysis of the homologous compounds such as caffeine, theobromine, and theophylline at the same time especially in the complex samples such as tea because the spectrums of these compounds are almost identical and prior powerful separation process is absent.

For optimization of MS conditions for the determination of caffeine, theobromine, and theophylline, 1.0 *μ*g/mL solution of each compound was infused individually into the mass spectrometer and its full mass spectrum was obtained. During the optimization process, it was observed that the signals obtained in the positive ionization mode were notably more intense than those in the negative ionization mode. Full-scan mass spectra of the three compounds were recorded, and the results showed that the highest peak corresponding to the protonated molecular ion has the mass-to-charge ratio of 195.19, 181.164, and 181.164 for caffeine, theobromine, and theophylline, respectively. The identification and quantitation of theobromine, theophylline, and caffeine in the tea samples were then accomplished using the SIR mode.

**Figure 1:**
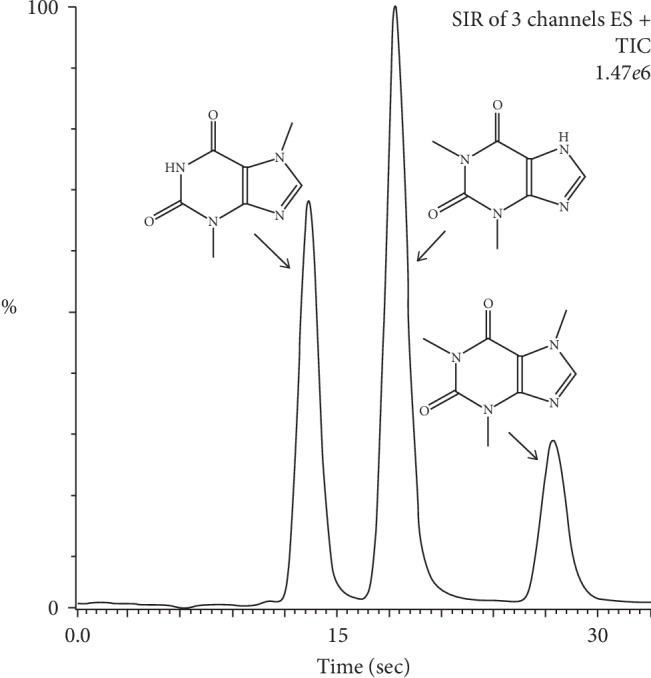
Chemical structures and chromatogram of the 1.0 *μ*g/mL theobromine, theophylline, and caffeine mixed standard solution at the optimized conditions.

The optimization procedure also included the verification of the influence of the capillary, cone, extractor and RF lens voltages, source, and desolvation temperatures and desolvation gas flow. It was observed that the best intensity of the three compounds was obtained at the conditions mentioned earlier in Table 2. At the optimized experimental conditions, the UHPLC-MS chromatogram obtained for 1.0 *μ*g/mL authentic sample of the three compounds is mentioned with the chemical structure of each compound in Figure 1. The optimization results of the MS method for caffeine, theobromine, and theophylline are given in Appendix A. in Supplementary data ([Supplementary-material supplementary-material-1]).

### 3.2. Validation of the Method

In order to evaluate the accuracy, sensitivity, specificity, and reproducibility of the analytical method, the proposed UHPLC-MS assay was validated in terms of linearity, LOD, LOQ, precision, and recovery.

#### 3.2.1. Calibration Curve, Linearity, LOD, LOQ, and Precision

Calibration curves of caffeine, theobromine, and theophylline were obtained (as shown in Appendix A. in Supplementary data) by injection of the standard solutions prepared in water. The developed method was found to be linear in the range of 0.08–5, 0.05–3, and 0.03–3 *μ*g/mL for caffeine, theobromine, and theophylline, respectively, the peak area being used for signal evaluation. However, the dynamic range observed for the three compounds was from 0.05 to 3.0 *μ*g/mL (as shown in Appendix A. Supplementary data). A good linearity was achieved in this concentration range with respect to the correlation coefficient (*R*^2^) of more than 0.9995 for the three compounds. The typical equation of calibration curve was *y* = 10746*x* + 377.15, *y* = 22200*x* +968.83, and *y* = 30507*x* + 3990.9 for caffeine, theobromine, and theophylline, respectively, where *y* is the peak area and *x* is the concentration of standard compound.

The LOD and LOQ values of the assay were determined based on the signal-to-noise criteria. The respective values of LOD were found to be 25, 15, and 10 ng/mL (S/N = 3) for caffeine, theobromine, and theophylline, respectively, while the respective values of LOQ were found to be 0.08, 0.05, and 0.03 *μ*g/mL (S/N = 10) for caffeine, theobromine, and theophylline, respectively.

#### 3.2.2. Repeatability, Reproducibility, and Ruggedness

Both repeatability and reproducibility of the proposed method were evaluated by intraday and interday precision, respectively. The standard mixtures (0.05, 0.1, 0.5, 1, 2, and 3 *μ*g/mL) were injected five times on the same day for intraday test and 25 times over five days for interday investigation. The percent relative standard deviation (%RSD) values for both the repeatability and reproducibility were 0.936–3.057%, 0.169–2.852%, and 0.086–1.971% for caffeine, theobromine, and theophylline standard samples, respectively. On the other hand, thirty real samples of tea were evaluated for repeatability by injecting each sample five times. The %RSD values were 0.807–3.137% and 0.251–4.082% for caffeine and theobromine, respectively, while theophylline was either found in trace or not detected in the tea samples. Thus, from these experiments, it could be concluded that the proposed assay can be fruitfully applied in the routine analysis of caffeine, theobromine, and theophylline present in various natural samples. All the quantitative results obtained for determination of the three compounds in thirty commercial tea samples are listed in Table 1. On the other side, the stability of the developed method against variations of the mobile phase composition, flow rate, and column temperature was checked in terms of the retention time of caffeine. The findings are reported in Table 4, and they confirmed the ruggedness of the developed method.

#### 3.2.3. Recovery Study

The recovery experiment was accomplished to estimate the applicability of the proposed method for the determination of all three analyzed xanthines. Tea sample number 5 was taken as a model for this test. The experiments were carried out by spiking the real sample extract with known concentration of the three components (caffeine, theobromine, and theophylline) at three levels: low, medium, and high (0.1, 1.0, and 3.0 μg/mL, respectively). The calculated recovery percentage values ranged between 82.4 and 96.6% with %RSD ranging from 0.95 to 3.72% (*n* = 3). These values reveal good extraction efficiencies. All recovery results are given in Table 5.

### 3.3. Analysis of Commercial Tea Samples

The applicability of the developed method for the routine quantitative analysis of xanthines in food was validated by the determination of caffeine, theobromine, and theophylline in tea samples. The method was fast, effective, sensitive, and reproducible for determination of these compounds in 30 commercial tea samples in which the analysis was accomplished within 30 seconds (more than 100 samples/h). Moreover, since the separation was achieved in isocratic elution mode with a mobile phase composed of 90% water and 10% acetonitrile, the proposed method can be considered as an inexpensive and environmentally friendly procedure, with the consumption of organic solvent being 3 mL per hour.

**Figure 2:**
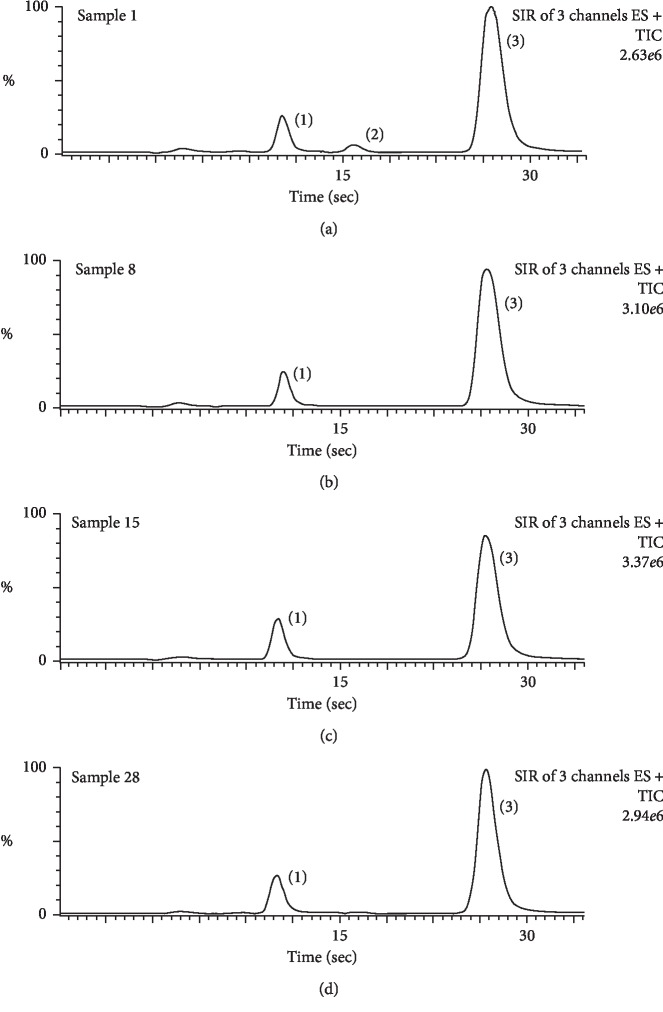
Representative UHPLC-MS chromatograms for some of the studied tea samples: (1) theobromine, (2) theophylline, and (3) caffeine.

The results showed that, in all the analyzed tea samples, the average content of caffeine was found to be 24.72 mg/g of tea, while the average theobromine content was found to be 1.05 mg/g of tea. On the other hand, theophylline was only found in sample no. 1 (0.14 mg/g) and in trace amounts in some tea samples, or not detected at all. These contents are in reasonable agreement with the previously published values of teas [50]. The highest concentration of caffeine (32.61 mg/g) and theobromine (2.72 mg/g) was found in sample no. 15. Very high concentrations of caffeine and theobromine were also found in sample nos. 24, 27, and 29, while caffeine and theobromine had the lowest concentrations in tea brands no. 1 and 23, respectively. All the outcomes of real samples are presented in Table 1. The results obviously show that the contents of theobromine are directly proportional to caffeine in each tea sample.  Figure 2 exhibits UHPLC-MS chromatograms for some selected tea samples. UHPLC-MS chromatograms for all of 30 tea samples are provided in Appendix B. Supplementary data.

## 4. Conclusion

A simple, fast, efficient, cost-effective, and eco-friendly UHPLC-MS method has been developed for the quantitative analysis of caffeine, theobromine, and theophylline components in commercial tea samples of various brands. Thirty tea samples were investigated, and the average contents of caffeine and theobromine were found to be 24.72 and 1.05 mg/g with LOD of 0.025 and 0.015 *μ*g/mL, respectively. With the exception of brand no. 1, theophylline was either found in traces in some tea samples or not detected in most of the analyzed samples. The maximum concentration of caffeine (32.61 mg/g) and theobromine (2.72 mg/g) was found in sample no. 15. High concentrations of caffeine and theobromine were also found in tea brand no. 27 (32.34 and 2.34 mg/g), brand no. 29 (31.84 and 2.10 mg/g), and brand no. 24 (29.70 and 1.99 mg/g). The extraction technique used in the analyses is very simple and mimics the traditional way for consumer tea preparation. Furthermore, the proposed analytical method reduced the consumption of time, samples, and solvents compared with the traditional ones. Both the quality parameters and the results obtained from this analysis method made the proposed UHPLC-MS method applicable for routine analysis of theobromine, theophylline, and caffeine with good precision and recovery.

## Figures and Tables

**Table 1:** The average content of theobromine, theophylline, and caffeine (mg/g ± %RSD) (*n* = 5) in commercial tea samples.

Sample no.	Concentration (mg/g ± %RSD)		
	Theobromine	Theophylline	Caffeine
1	0.27 ± 0.25	0.14 ± 2.61	13.30 ± 1.68
2	1.22 ± 2.66	ND	27.58 ± 1.63
3	0.44 ± 0.55	Traces	17.06 ± 1.24
4	0.41 ± 2.64	Traces	23.15 ± 2.32
5	1.16 ± 2.84	ND	27.95 ± 2.79
6	0.55 ± 1.55	ND	23.29 ± 2.05
7	0.63 ± 0.47	ND	18.37 ± 1.99
8	0.99 ± 4.08	ND	25.90 ± 2.58
9	0.63 ± 0.77	ND	20.96 ± 2.51
10	1.39 ± 3.39	ND	29.16 ± 2.45
11	0.86 ± 2.11	ND	23.48 ± 1.17
12	0.85 ± 3.03	Traces	26.32 ± 2.32
13	1.16 ± 0.95	Traces	25.32 ± 0.83
14	1.76 ± 2.40	ND	27.33 ± 1.67
15	2.72 ± 1.69	ND	32.61 ± 0.81
16	1.18 ± 2.26	ND	26.99 ± 1.54
17	1.73 ± 3.23	ND	26.63 ± 3.02
18	1.15 ± 1.87	ND	27.58 ± 1.23
19	1.01 ± 3.36	ND	25.83 ± 3.14
20	0.57 ± 2.66	ND	20.09 ± 2.56
21	0.44 ± 1.13	Traces	22.55 ± 1.92
22	0.50 ± 1.31	Traces	22.51 ± 3.03
23	0.20 ± 0.48	ND	16.11 ± 0.81
24	1.99 ± 2.97	ND	29.70 ± 3.02
25	1.10 ± 2.51	ND	21.06 ± 2.98
26	0.86 ± 2.45	ND	30.30 ± 1.67
27	2.34 ± 2.00	ND	32.34 ± 2.78
28	0.49 ± 1.51	ND	22.33 ± 2.86
29	2.10 ± 3.30	ND	31.84 ± 2.78
30	0.72 ± 1.19	ND	23.84 ± 1.85

**Table 2:** Optimum HPLC and MS conditions for determination of theobromine, theophylline, and caffeine.

Items	Parameters
*HPLC conditions*	
Column	Nucleodur C18 polar Tec. (50 × 2 mm i.d.; 1.8 *μ*m particle sizes)
Mobile phase	Acetonitrile: water (10 : 90, v/v) with 1.0% formic acid
Flow rate	0.5 mL/min
Column temperature	70°C
Injection volume	5.0 *μ*L

*MS conditions*	
MS mode	ESI^+^ (SIM mode)
Capillary (KV)	3.5
Extractor (V)	2
RF lens (V)	0
Cone voltage (V)	35
Source temperature (°C)	120
Desolvation temperature (°C)	300
Desolvation gas flow (L/h)	600
Cone gas flow (L/h)	60

**Table 3:** Comparison of the proposed method with some of the recently reported HPLC methods for the determination of caffeine.

Ref.	Analytical column (length mm × i.d. mm, particle size *μ*m)	Elution type	Flow rate (mL/min)	Retention time (min)^*∗*^
[1]	Inertsil ODS-3v (250 × 4.6, 5)	Isocratic	1.5	5.82
[2]	PartiSphere 5 C18 (250 × 4.6, 5)	Gradient	1.0	10.95
[10]	Fused core Kinetex C18 (100 × 4.6, 2.6)	Gradient	2.2	1.79
[13]	Poroshell 120 EC-C18 (50 × 4.6, 2.7)	Isocratic	0.5	5.0
[15]	BDS HypersilGold C18 (250 × 4.6, 5)	Isocratic	1.4	8.2
[15]	Merck monolithic Rp-18 e (100 × 4.6)	Isocratic	1.4	2.6
[19]	Chromolith SpeedRod (50 × 4.6)	Isocratic	3.0	0.68
[20]	Hexyl methacrylate monolith (150 × 0.53)	Isocratic	0.041	1.16
[20]	Betasil C18 (150 × 4.6, 3)	Isocratic	0.5	15.41
[37]	Kinetex C18 (150 × 4.6, 5)	Gradient	1.0	11.71
[38]	UPLC C18 BEH (50 × 2.1, 1.7)	Isocratic	0.7	2.52
[39]	Eclipse XDB-C8 (150 × 4.6, 5)	Isocratic	1.0	2.1
[40]	Agilent TC-C18 (250 × 4.6, 5)	Gradient	0.75	38.0
[41]	BEH HILIC C18 (50 × 2.1, 1.7)	Isocratic	0.25	1.15
[42]	Eclipse C18 (250 × 4, 5)	Gradient	1.0	10.0
[43]	Scherzo SS-C18 (150 × 4.6, 3)	Gradient	0.4	12.6
[44]	Diol-HILIC (150 × 3.0, 5)	Gradient	0.3	4.1
[45]	XBridge C18 (150 × 4.6, 3.5)	Isocratic	1.0	7.16
[46]	Gemini C18 (150 × 4.6, 5)	Gradient	1.0	18.6
[47]	BEH C18 (100 × 2.1, 1.7)	Gradient	0.25	4.24
[48]	Kinetex C18 (100 × 2.1, 2.6)	Gradient	0.2	3.6
[48]	HypersilGold (50 × 2.1, 1.9)	Gradient	0.2	1.9
[49]	Titan C18 (100 × 2.1, 1.9)	Gradient	0.3	6.81
This work	Nucleodur C18 polar Tec. (50 × 2, 1.8)	Isocratic	0.5	0.46

^*∗*^Retention time for caffeine (min). Since it was not mentioned in some studies, the retention time of caffeine was carefully estimated from the separation chromatogram.

**Table 4:** Ruggedness study of the proposed method.

Chromatographic conditions	Retention time of caffeine *t*_R_ (sec) ± %RSD (*n* = 3)	Percentage change
Optimum conditions	28 ± 0.62	—

Effect of mobile phase flow rate		
0.55 mL/min	23 ± 0.49	18
0.45 mL/min	35 ± 0.40	25

Effect of mobile phase composition		
ACN: H_2_O (15 : 85, v/v)	24 ± 0.33	14
ACN: H_2_O (5 : 95, v/v)	33 ± 0.56	18

Effect of column temperature		
75°C	26 ± 0.51	7
65°C	31 ± 0.37	11

**Table 5:** Recovery and repeatability of the proposed analytical method at three different spiking levels.

Concentration (*μ*g/mL)	Mean recovery (%) ± %RSD (*n* = 3)		
	Theobromine	Theophylline	Caffeine
0.1	86.9 ± 3.6	83.4 ± 2.2	82.4 ± 3.7
1.0	95.7 ± 1.6	96.6 ± 1.0	93.7 ± 1.1
3.0	95.7 ± 1.6	94.1 ± 1.6	96.2 ± 2.3

## Data Availability

The data used to support the findings of this study and the brand names of the commercial tea samples are available from the corresponding author upon request.

## Supplementary Materials

Supplementary Materials The supporting information file includes additional results and information as described in the text of the main article, including the optimization results of MS method for caffeine, theobromine, and theophylline and the UHPLC-MS chromatograms for all tea samples.
