# Supplementary material for: Rapid and Sensitive Determination of Methylxanthines in Commercial Brands of Tea Using Ultra-High-Performance Liquid Chromatography-Mass Spectrometry
Source: Int J Anal Chem. 2019 Nov 3;2019:2926580. doi: 10.1155/2019/2926580 (PMC6875304; doi:10.1155/2019/2926580)
Supplement: Supplementary Materials — The supporting information file includes additional results and information as described in the text of the main article, including the optimization results of MS method for caffeine, theobromine, and theophylline and the UHPLC-MS chromatograms for all tea samples. [file 2926580.f1.docx]

**Supplementary Data**

**Rapid and sensitive determination of methylxanthines in commercial brands of tea using ultra-high performance liquid chromatography-mass spectrometry**

Ahmad Aqel^1,*^, Ahmed Almulla^1^, Asma’a Al-Rifai^2,*^, Saikh M. Wabaidur^1^, Zeid A. ALOthman^1^, Ahmed-Yacine Badjah-Hadj-Ahmed^1^

^1^Department of Chemistry, College of Science, King Saud University, P.O. Box 2455, Riyadh 11451, Saudi Arabia

^2^Department of Chemistry, College of Science, Princess Nourah bint Abdulrahman University, Riyadh, Saudi Arabia

^*^ Corresponding Author:

aifseisi@ksu.edu.sa (A. Aqel)

asmaaaalrifai@pnu.edu.sa (A. Al-Rifai)

Telephone: +966 114674198, Fax: +96614675992

**Summary**

This supporting information file includes additional results and information as described in the text of the main article. Including:

Appendix A. The optimization results of MS method for caffeine, theobromine and theophylline

Appendix B. UHPLC-MS chromatograms for all of 30 tea samples

Appendix A (Fig. S1-S3)


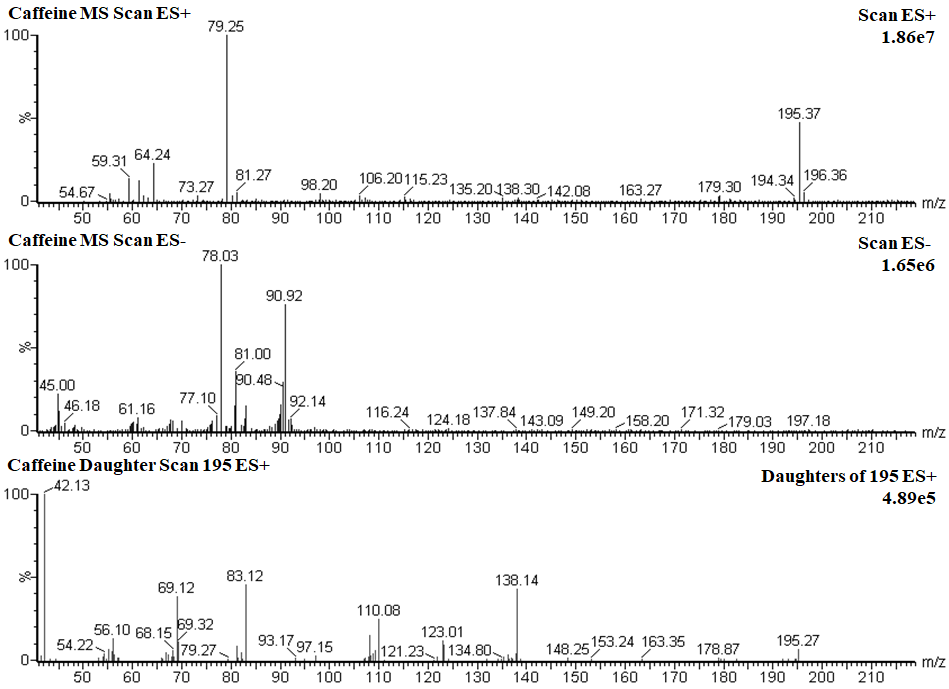


Fig. S1 Optimization results of MS method for caffeine


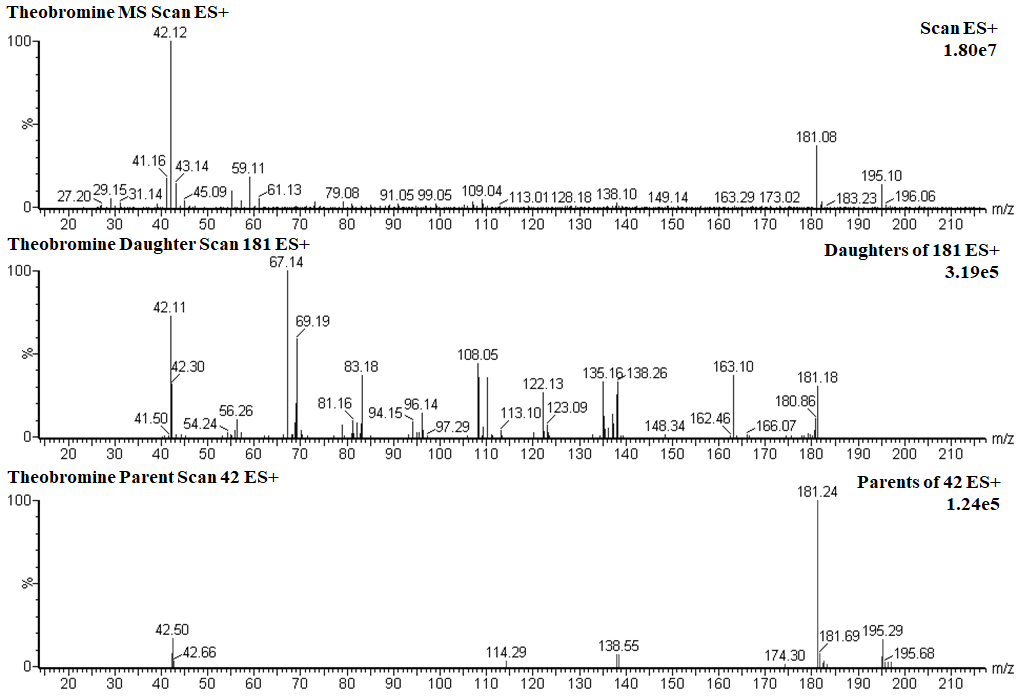


Fig. S2 Optimization results of MS method for theobromine


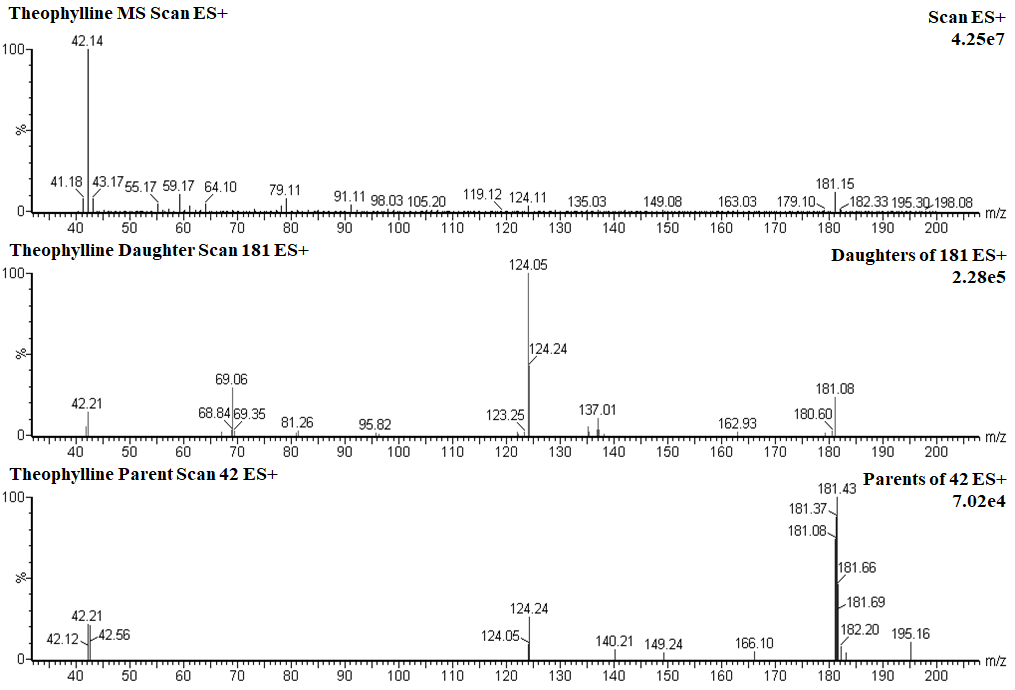


Fig. S3 Optimization results of MS method for theophylline


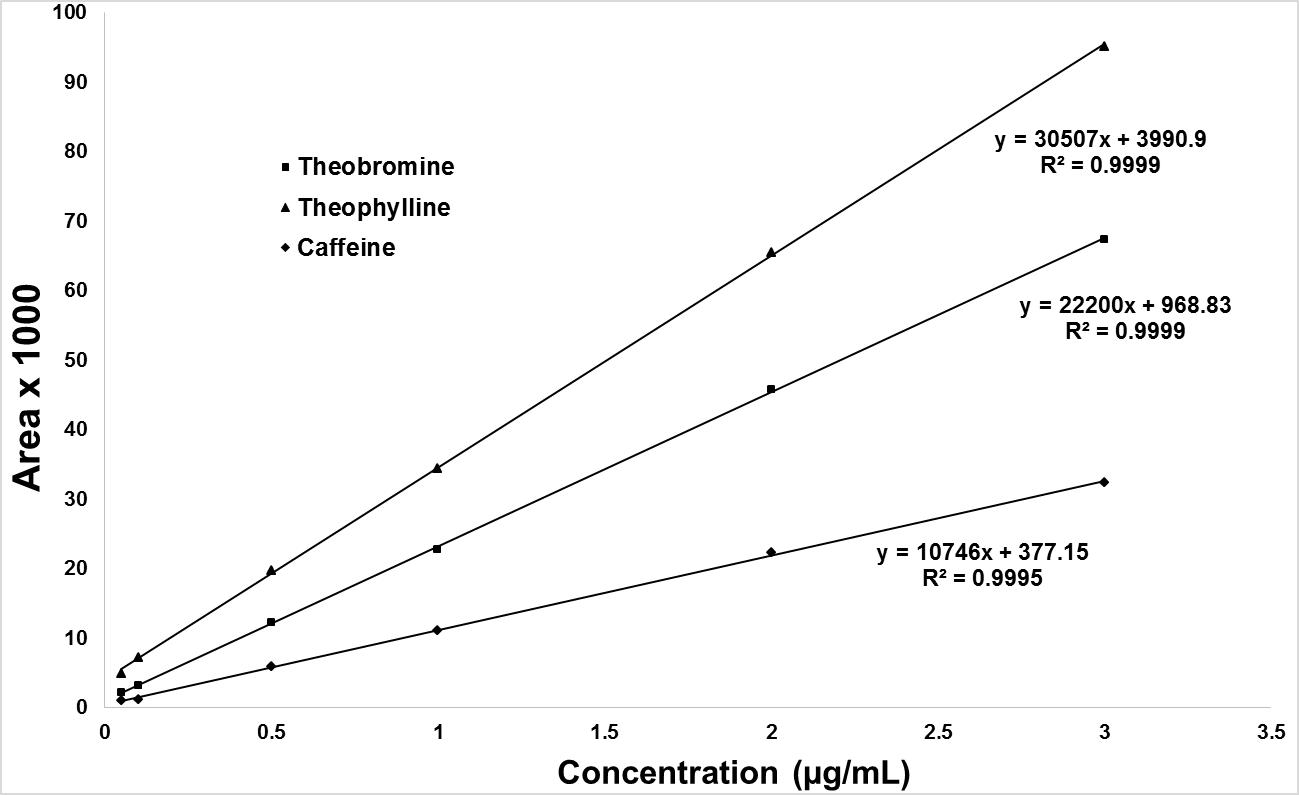


Fig. S4 Peak area vs. concentration plots of theobromine, theophylline and caffeine

Appendix B (Fig. S4-S33)


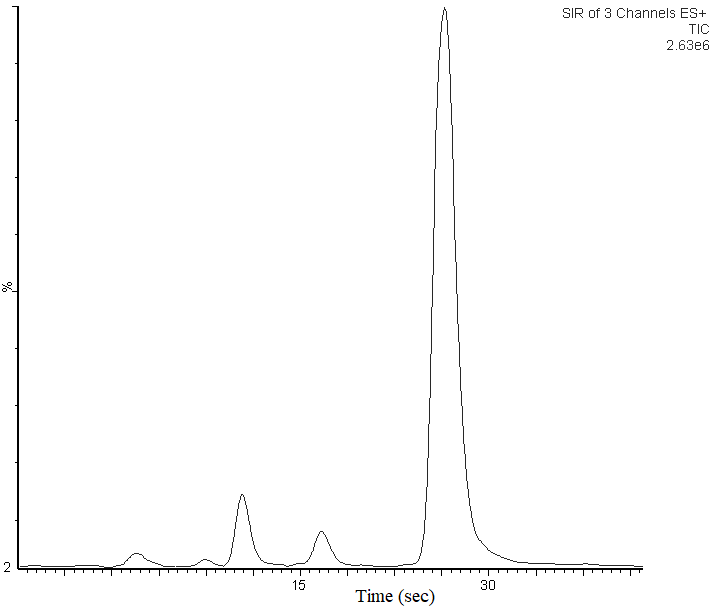


Fig. S5 UHPLC-MS chromatogram for sample no. 1


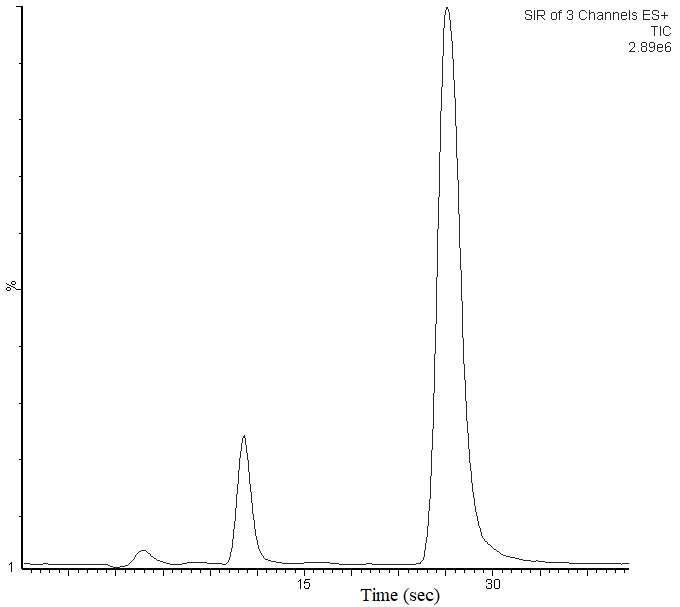


Fig. S6 UHPLC-MS chromatogram for sample no. 2


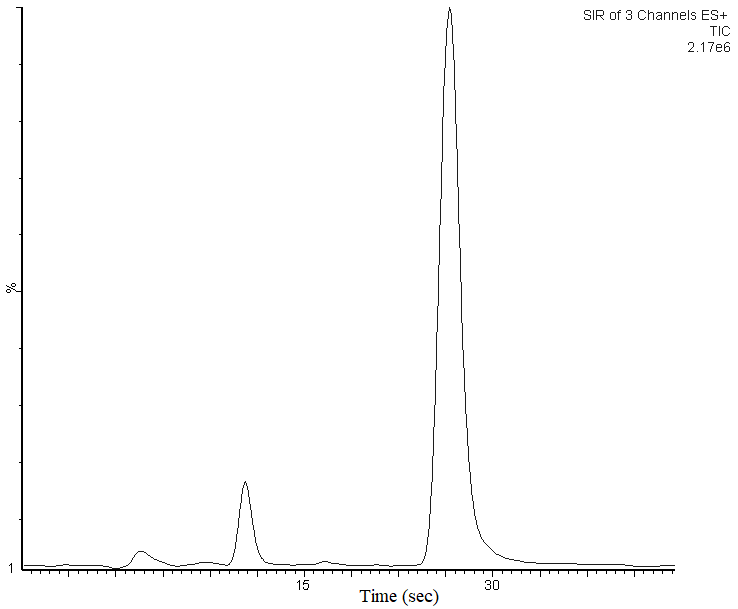


Fig. S7 UHPLC-MS chromatogram for sample no. 3


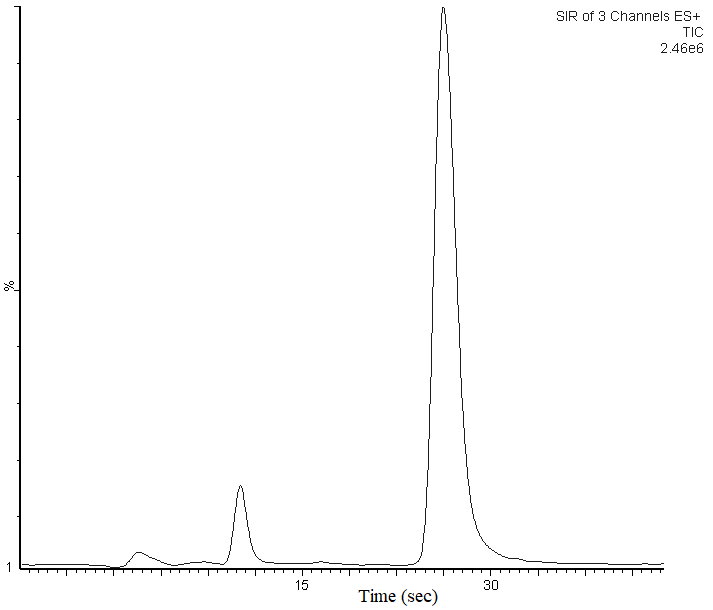


Fig. S8 UHPLC-MS chromatogram for sample no. 4


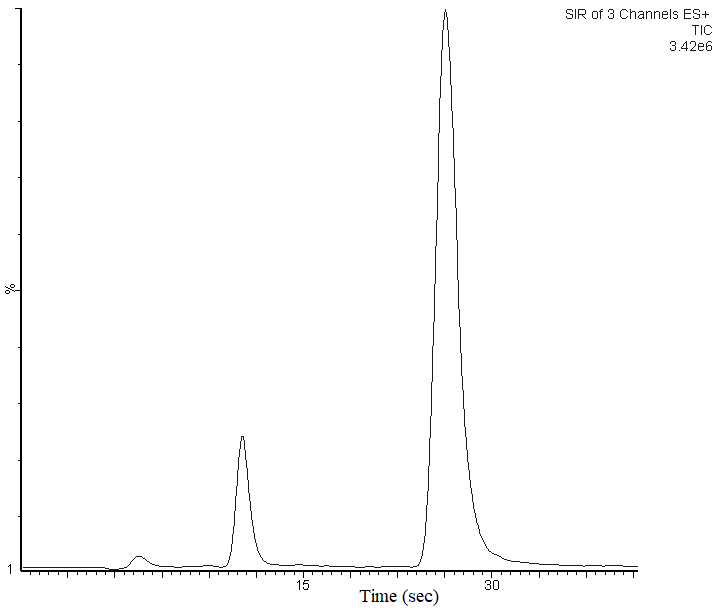


Fig. S9 UHPLC-MS chromatogram for sample no. 5


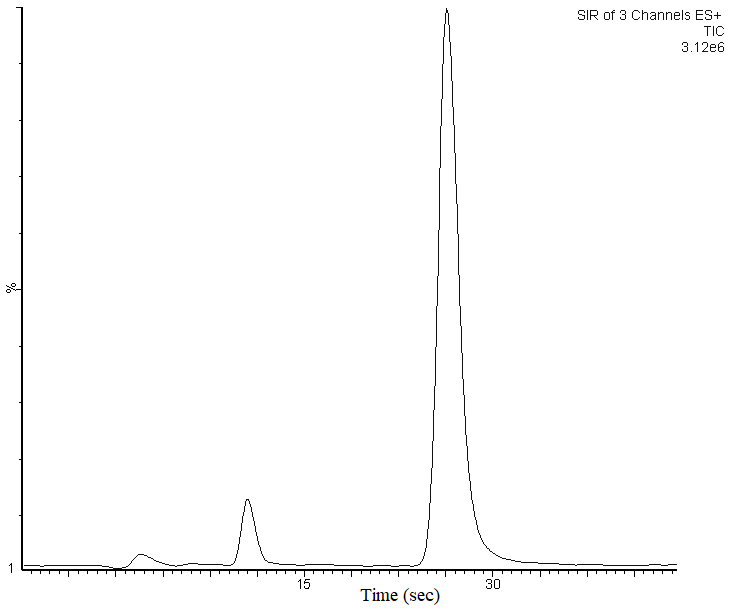


Fig. S10 UHPLC-MS chromatogram for sample no. 6


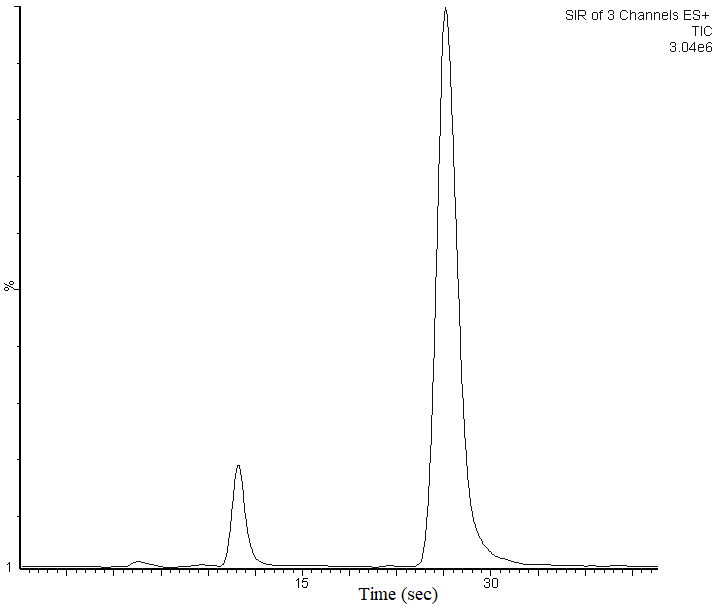


Fig. S11 UHPLC-MS chromatogram for sample no. 7


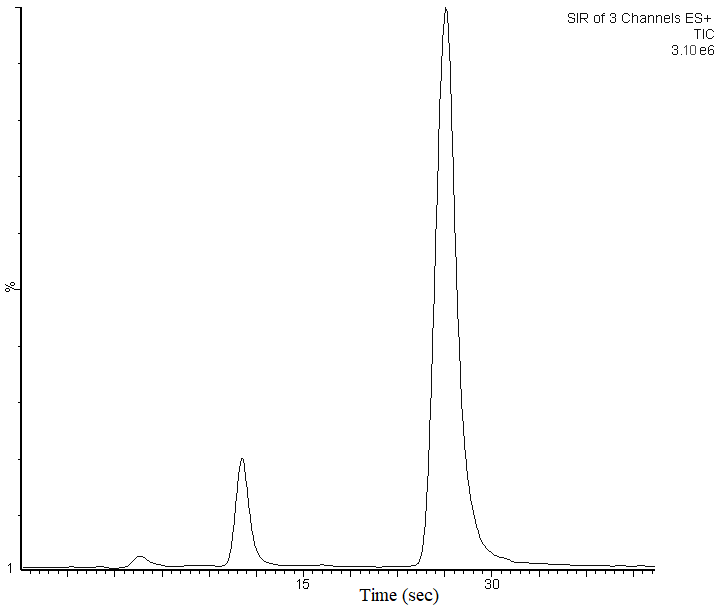


Fig. S12 UHPLC-MS chromatogram for sample no. 8


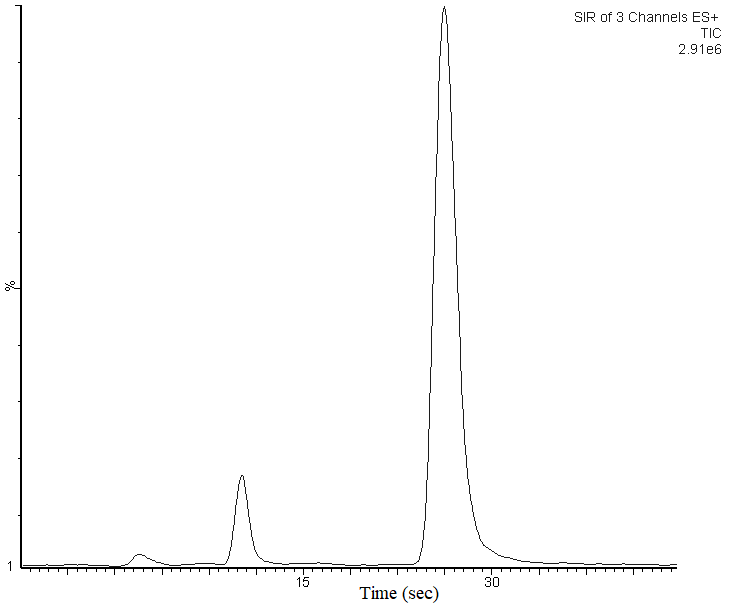


Fig. S13 UHPLC-MS chromatogram for sample no. 9


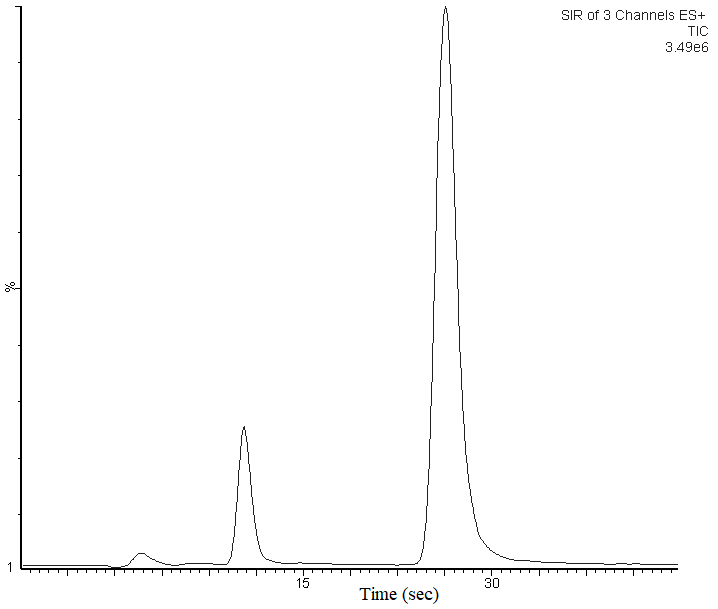


Fig. S14 UHPLC-MS chromatogram for sample no. 10


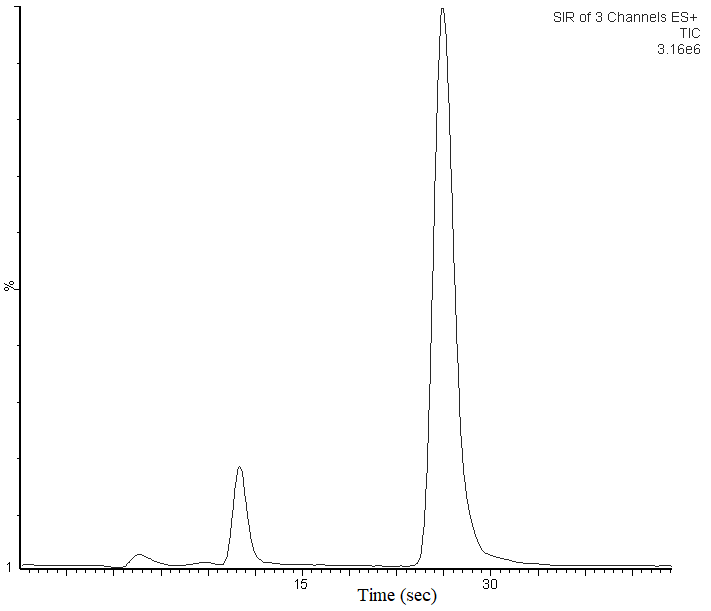


Fig. S15 UHPLC-MS chromatogram for sample no. 11


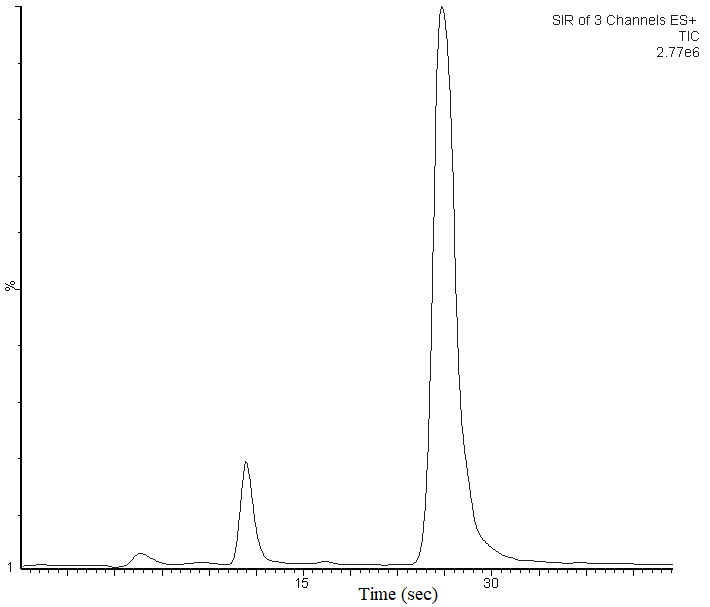


Fig. S16 UHPLC-MS chromatogram for sample no. 12


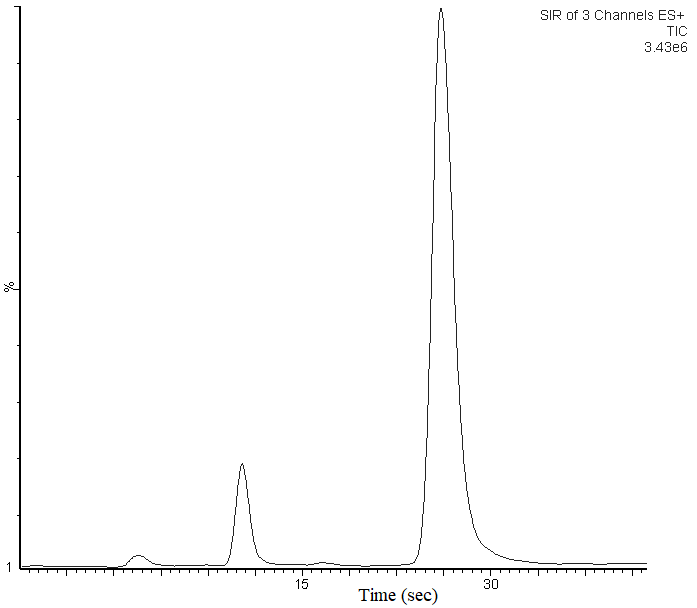


Fig. S17 UHPLC-MS chromatogram for sample no. 13


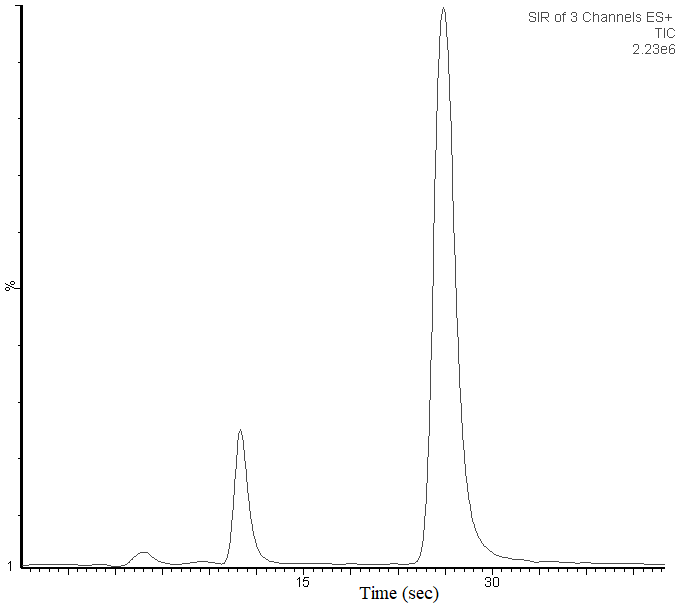


Fig. S18 UHPLC-MS chromatogram for sample no. 14


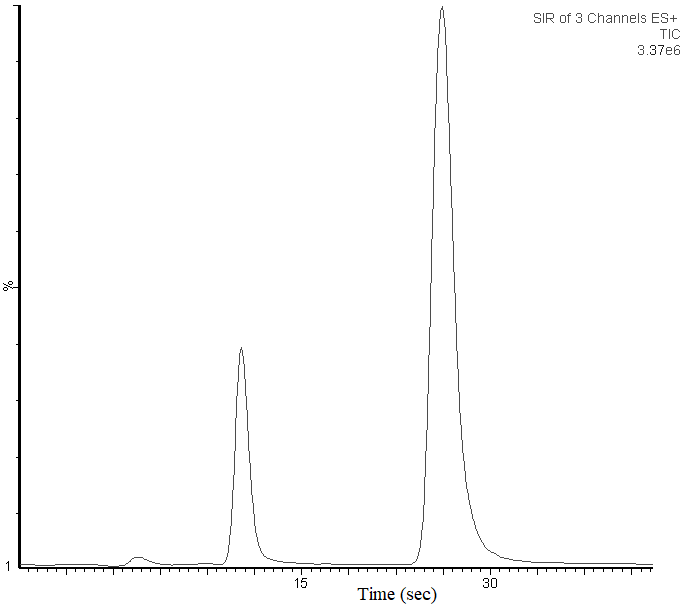


Fig. S19 UHPLC-MS chromatogram for sample no. 15


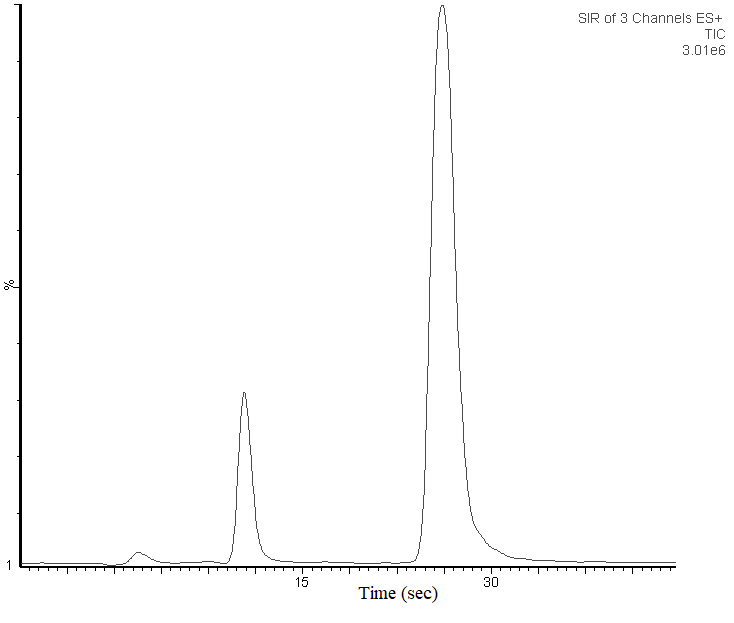


Fig. S20 UHPLC-MS chromatogram for sample no. 16


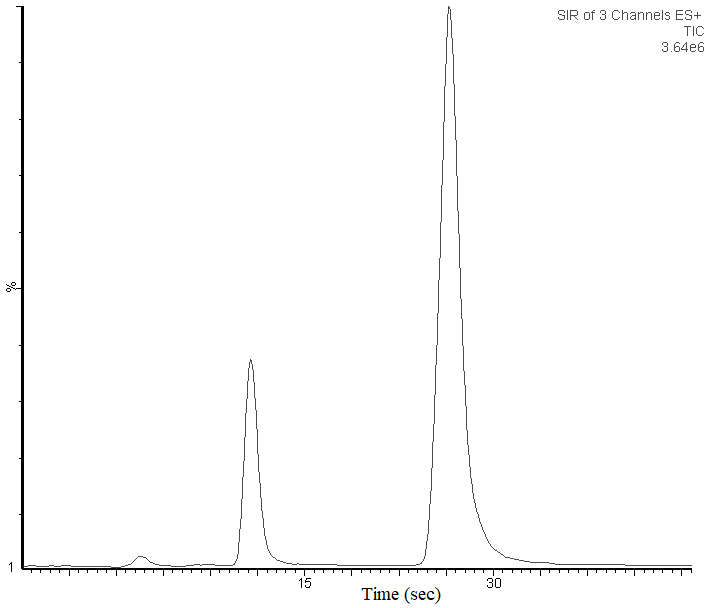


Fig. S21 UHPLC-MS chromatogram for sample no. 17


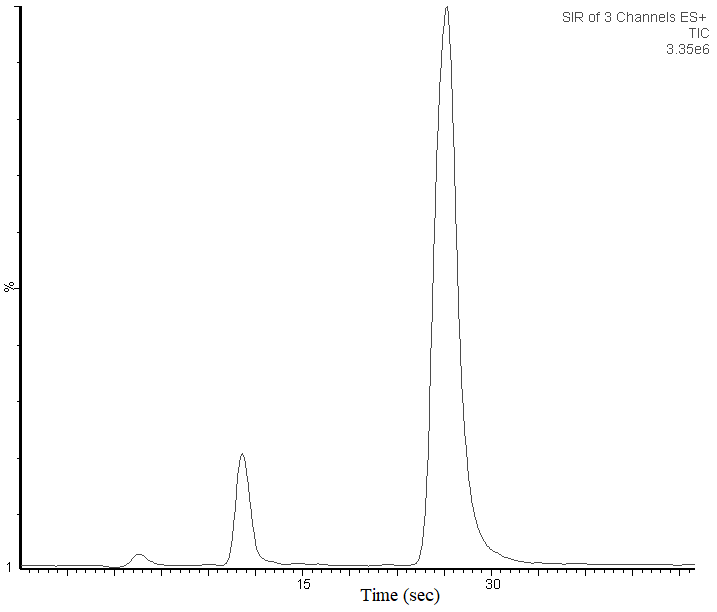


Fig. S22 UHPLC-MS chromatogram for sample no. 18


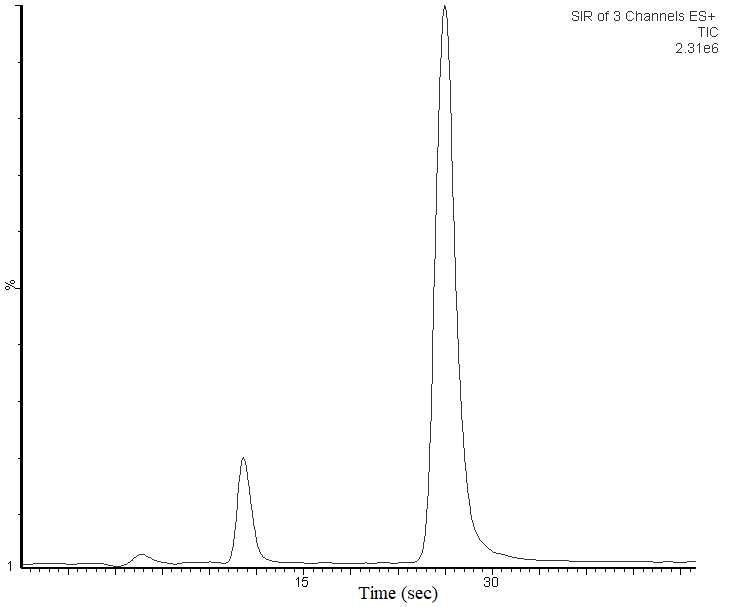


Fig. S23 UHPLC-MS chromatogram for sample no. 19


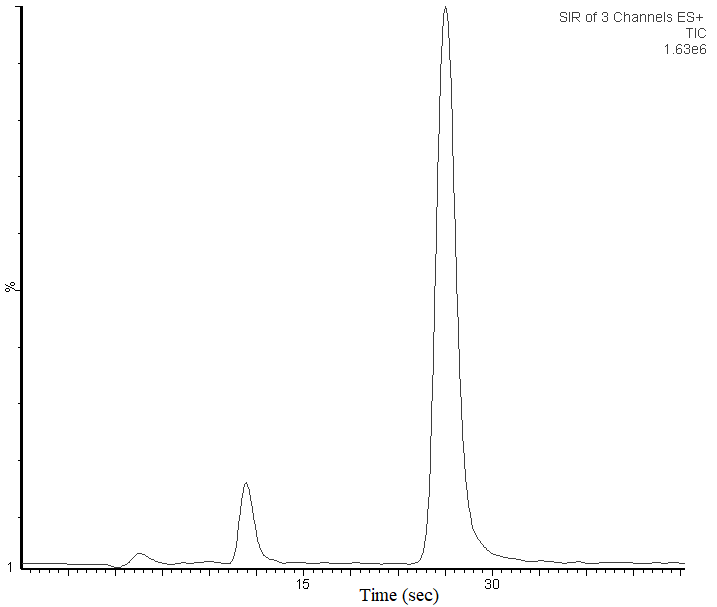


Fig. S24 UHPLC-MS chromatogram for sample no. 20


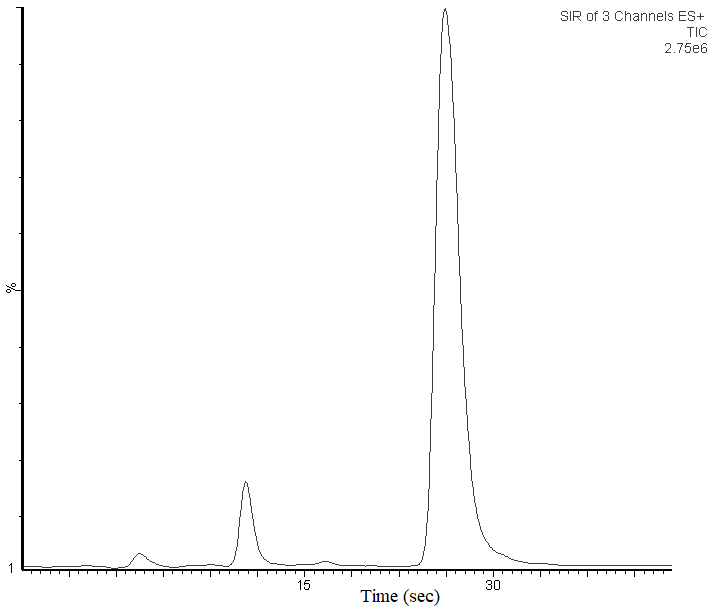


Fig. S25 UHPLC-MS chromatogram for sample no. 21


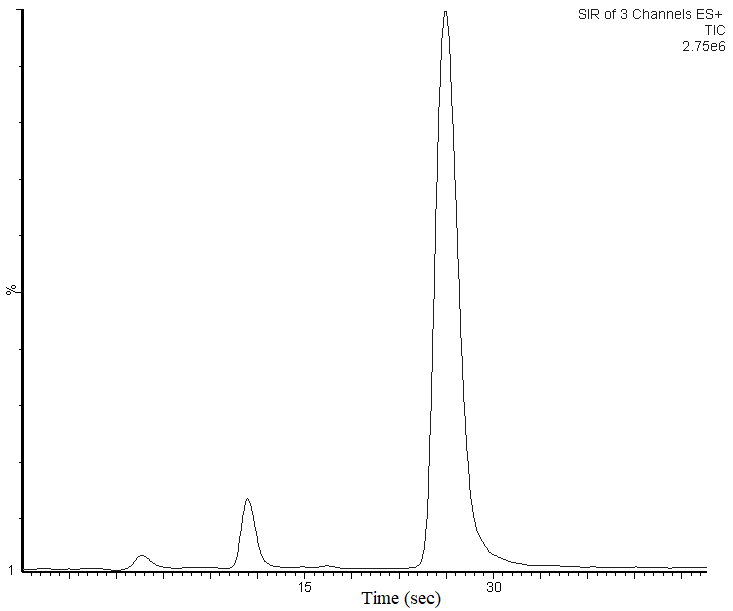


Fig. S26 UHPLC-MS chromatogram for sample no. 22


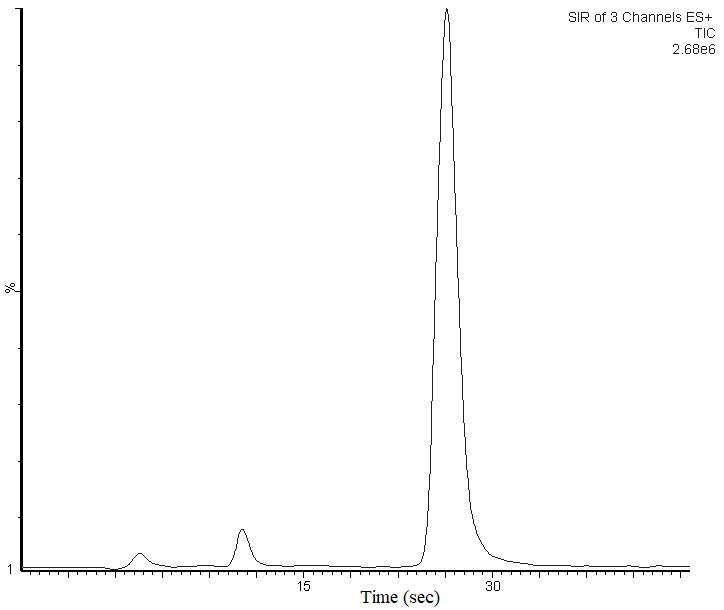


Fig. S27 UHPLC-MS chromatogram for sample no. 23


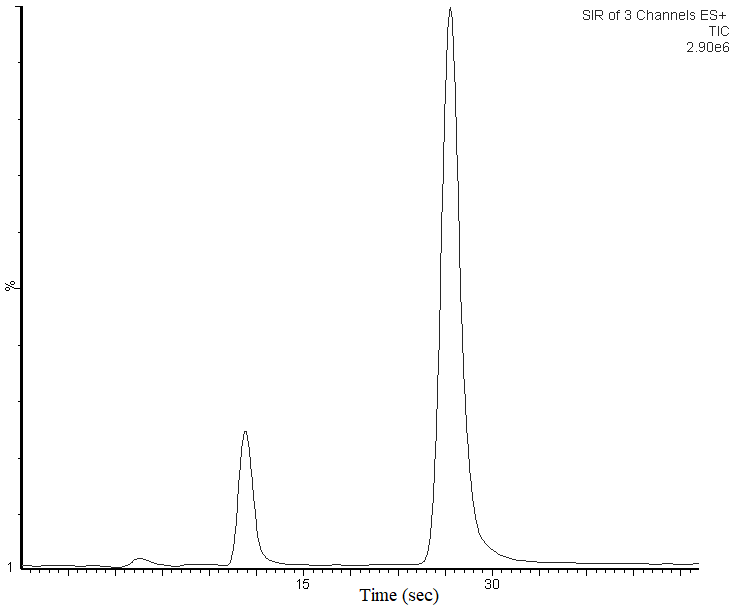


Fig. S28 UHPLC-MS chromatogram for sample no. 24


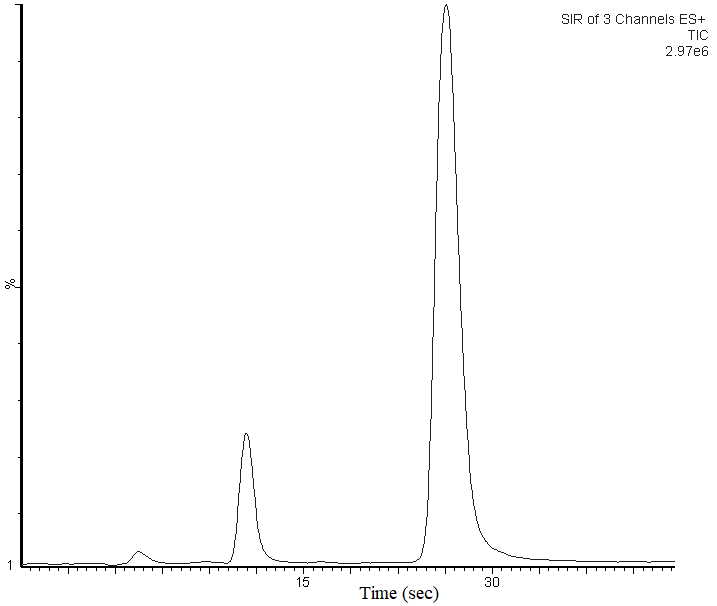


Fig. S29 UHPLC-MS chromatogram for sample no. 25


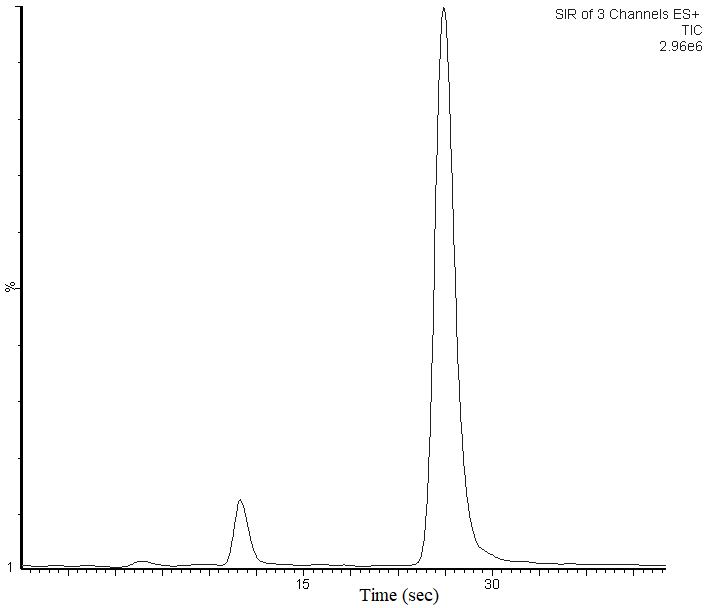


Fig. S30 UHPLC-MS chromatogram for sample no. 26


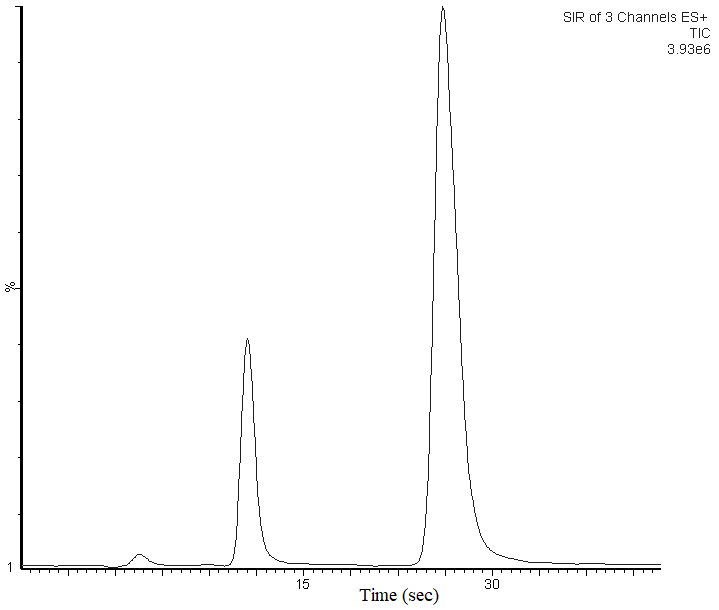


Fig. S31 UHPLC-MS chromatogram for sample no. 27


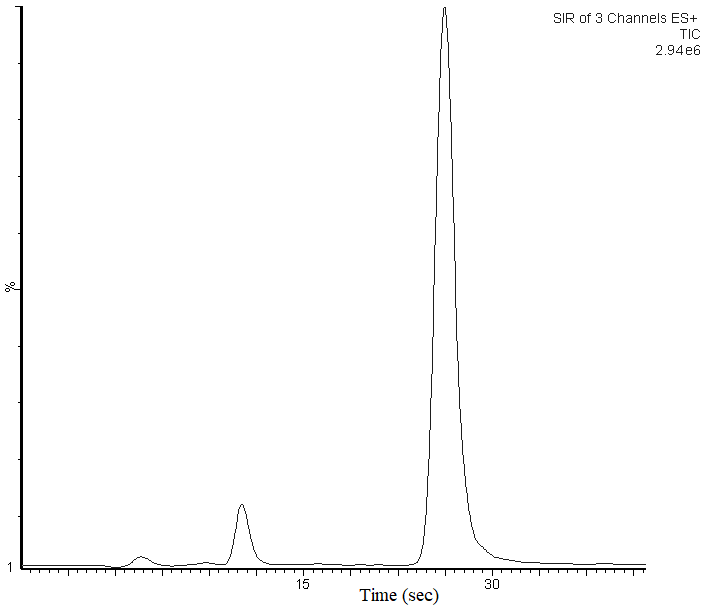


Fig. S32 UHPLC-MS chromatogram for sample no. 28


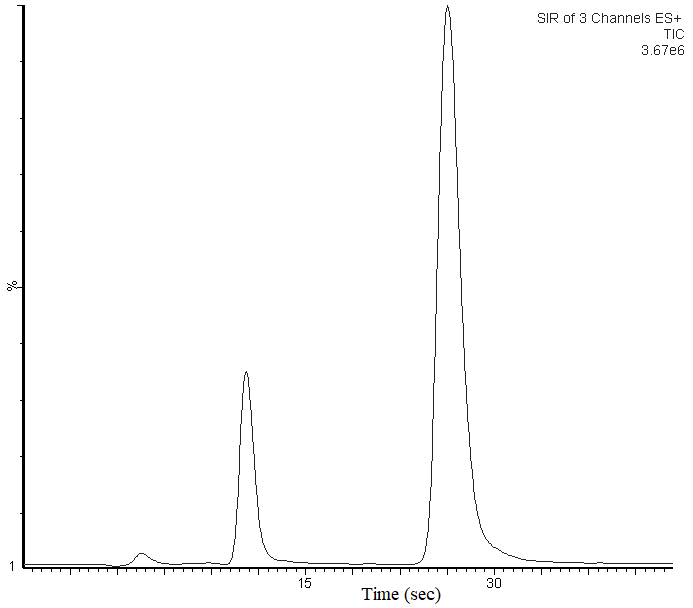


Fig. S33 UHPLC-MS chromatogram for sample no. 29


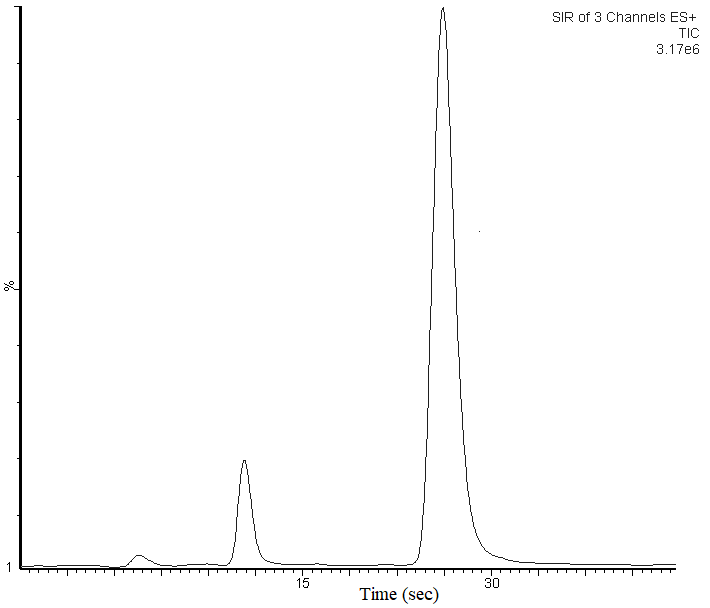


Fig. S34 UHPLC-MS chromatogram for sample no. 30
